# The Association Between Cadmium Exposure and Gestational Diabetes Mellitus: A Systematic Review and Meta-Analysis

**DOI:** 10.3389/fpubh.2021.555539

**Published:** 2022-02-10

**Authors:** Yu Lin, Ting Li, Jiangbo Xiao, Kaipeng Xie, Zhonghua Shi

**Affiliations:** ^1^Department of Obstetrics and Gynecology, Nanjing Maternity and Child Health Care Hospital, Women's Hospital of Nanjing Medical University, Nanjing, China; ^2^Department of Public Health, Nanjing Maternity and Child Health Care Hospital, Women's Hospital of Nanjing Medical University, Nanjing, China; ^3^Department of Women Health Care, Nanjing Maternity and Child Health Care Hospital, Women's Hospital of Nanjing Medical University, Nanjing, China

**Keywords:** cadmium, gestational diabetes mellitus, meta-analysis, metal, systematic review

## Abstract

**Objective:**

Several studies have evaluated the association of cadmium exposure with the risk of gestational diabetes mellitus (GDM). However, the findings among these studies have been inconsistent. To further investigate the relationship, we carried out a meta-analysis to clarify the relationship between cadmium exposure and GDM risk.

**Methods:**

Five databases (Scopus, PubMed, Web of Science, Cochrane, and CNKI) were searched for eligible studies until September 09, 2021. The quality of eligible studies was evaluated using the Newcastle–Ottawa quality assessment scale (NOS). The summary odds ratios (ORs) and 95% confidence intervals (CIs) were calculated by random-effects models due to high heterogeneity. Sensitivity analysis was performed to explore the robustness of the results. Publication bias was evaluated by Egger's test and Begg's test. We also conducted meta-regression analysis and subgroup analysis to assess the potential sources of heterogeneity.

**Results:**

A total of 10 studies with 32,000 participants related to our issue were included. Comparing the highest vs. lowest categories of cadmium exposure, no significant association was observed between cadmium exposure and the risk of GDM (OR = 1.16, 95% CI = 0.92–1.46, and *P* = 0.206). No publication bias was found in Begg's and Egger's tests (all *P* > 0.05). Meta-regression suggested that publication year was the potentially heterogeneous source (*P* = 0.034). Subgroup analysis of publication year showed that the OR of studies before the year of 2016 was 4.05 (95% CI = 1.87–8.76, *P* < 0.001), and prospective cohort studies showed a borderline increased GDM risk (OR = 1.15, 95% CI = 0.99–1.33, and *P* = 0.061).

**Conclusion:**

Our results indicated no significant association between cadmium exposure and GDM risk. Further high-quality prospective studies, especially those using standard analytic methods for cadmium exposure, are warranted to confirm the results.

## Introduction

Gestational diabetes mellitus (GDM) is the initial occurrence of abnormal glucose tolerance during pregnancy and has a worldwide prevalence ranging from 1 to >30%, indicating that GDM is a relatively prevalent disease during pregnancy (1). The maternal and fetal consequences of GDM have been well-explored. The common complications of GDM include preeclampsia, polyhydramnios, operative delivery, shoulder dystocia, fetal overgrowth, neonatal hypoglycemia, jaundice, and perinatal mortality (2). Moreover, mothers and their offspring have a tendency to develop type 2 diabetes and obesity in the long term (3).

Epidemiological studies have confirmed a series of risk factors contributing to GDM, such as advanced maternal age, geography, ethnicity, and lifestyle factors. However, emerging risk factors, such as environmental pollutants and endocrine disruptors, have also attracted more attention (4, 5). As a result, it is vital to take emerging risk factors into account for the prevention and management of GDM. Cadmium, recognized as a toxic heavy metal distributed widely in our environment, has a large number of negative effects on humans. An unhealthy diet and tobacco smoking are the major sources of cadmium exposure in humans. Absorbed cadmium accumulates mainly in the kidneys and is ultimately excreted through the urine (6). The carcinogenicity and damage to the kidneys caused by cadmium have been reported in some studies (7, 8). Moreover, there is abundant evidence indicating a strong association between cadmium exposure and type 2 diabetes. The diabetogenic effect of cadmium may be involved in the dysfunction of insulin secretion from glucose-stimulated β-cells (9, 10).

Considering the increased risk of diabetes resulting from cadmium, we hypothesized that cadmium exposure may also increase the risk of GDM by similar mechanisms. Several studies found significant evidence to prove the relationship between cadmium and the risk of GDM. However, others found negative results, which may be due to differences in study populations, regions, sample types, and study methods. To explore the association more deeply, we conducted a meta-analysis to synthesize the data from existing studies and to analyze the likely relationship between cadmium and the risk of GDM.

## Methods

### Search Strategies

A systematic search for published studies that reported the relationship between cadmium and GDM risk was conducted using the following five databases from the date of database inceptions to September 09, 2021: Scopus, PubMed, Web of Science, Cochrane, and Chinese National Knowledge Infrastructure (CNKI). As for PubMed, the search was designed to seek articles that reported the effect of cadmium on GDM risk in English databases by combining MeSH terms and free-text terms: (((((((Diabetes, Gestational[MeSH Terms]) OR Diabetes, Pregnancy-Induced) OR Diabetes, Pregnancy Induced) OR Pregnancy-Induced Diabetes) OR Gestational Diabetes) OR Diabetes Mellitus, Gestational) OR Gestational Diabetes Mellitus) AND ((metals [MeSH Terms]) OR cadmium). In other English databases, the keywords used for the search were (((Gestational Diabetes) OR Pregnancy-Induced Diabetes) OR Gestational Diabetes Mellitus) AND (metals OR cadmium). Regarding the CNKI, we used the terms gestational diabetes mellitus, metals, and cadmium in Chinese and then combined the terms “gestational diabetes mellitus” and “metals or cadmium” to search for potential articles. The search strategy of each database is presented in Supplementary Figure 1.

### Inclusion and Exclusion Criteria

The participants, interventions, comparison, outcomes, and study design (PICOS) strategy was used to guide our eligibility criteria (Supplementary Table 1). Studies were included if they reported the association between cadmium exposure and the risk of GDM. The criteria required for eligibility were as follows: (1) The diagnostic criteria of GDM were cautiously defined according to standard criteria; (2) proper and reasonable measurements were used to describe the cadmium concentrations in pregnant women; (3) cadmium exposures in pregnancy or prior to pregnancy were all considered; and (4) the odds ratios (ORs) or relative ratios (RRs) and 95% confidence intervals (CIs) or other statistics could be inverted into odds ratios and 95% CIs. The exclusion criteria were as follows: (1) duplicated reports; (2) animal studies, reviews, news, and abstracts; (3) ecological studies; (4) inadequate data or incorrect statistical analysis; and (5) articles that focused on diabetes rather than GDM.

### Data Extraction and Quality Assessment

Two investigators independently extracted the following information regarding the eligible studies: (1) First author and timing of publication; (2) time period and location of the study; (3) study design; (4) sample type; (5) time of sample collection; (6) sample size; (7) diagnostic criteria for GDM; (8) concentration of cadmium; (9) covariates adjusted; and (10) OR/RR and 95% CI. The OR/RR and 95% CI were extracted from the adjusted models. When divergence occurred, the two investigators asked the mentor. Among the eligible studies, a study by Tomoko Oguri and his colleagues included nulliparous and parous women. As a result, we included them as two studies in our meta-analysis. Jia et al. used the prevalence ratios (PRs) to describe the risk. Based on the previous study (11), we also used the PRs. The quality of each study was assessed according to the Newcastle–Ottawa quality assessment scale (NOS) and divided into different grades based on their scores as follows: (1) a score ≤ 3 was regarded as low quality; (2) a score of 4–6 was regarded as intermediate quality; and (3) a score ≥ 7 was regarded as high quality.

### Statistical Analysis

Heterogeneity among the eligible studies was measured by Cochran's *Q*-test and inconsistency index (*I*^2^). Pooled OR and 95% CI were calculated using the fixed-effects model if the heterogeneity test was not significant (*P* ≥ 0.05). Otherwise, the random-effects model was adopted. Sensitivity analyses were performed by excluding studies one after the other to estimate the stability of the pooled findings. Begg's and Egger's tests were performed to evaluate potential publication bias. Multivariate meta-regression analysis was applied to assess the possible sources of heterogeneity. Subgroup analysis was performed by publication year, geographic region, study design, diagnosis criteria, sample type, sample size, NOS score, and time of collection. All statistical analyses were two-sided and carried out by StataSE 12.0 (Stata Corp LP, College Station, Texas, USA). *P* < 0.05 indicated statistical significance.

## Results

### Search Results and Study Characteristics

**Figure 1:**
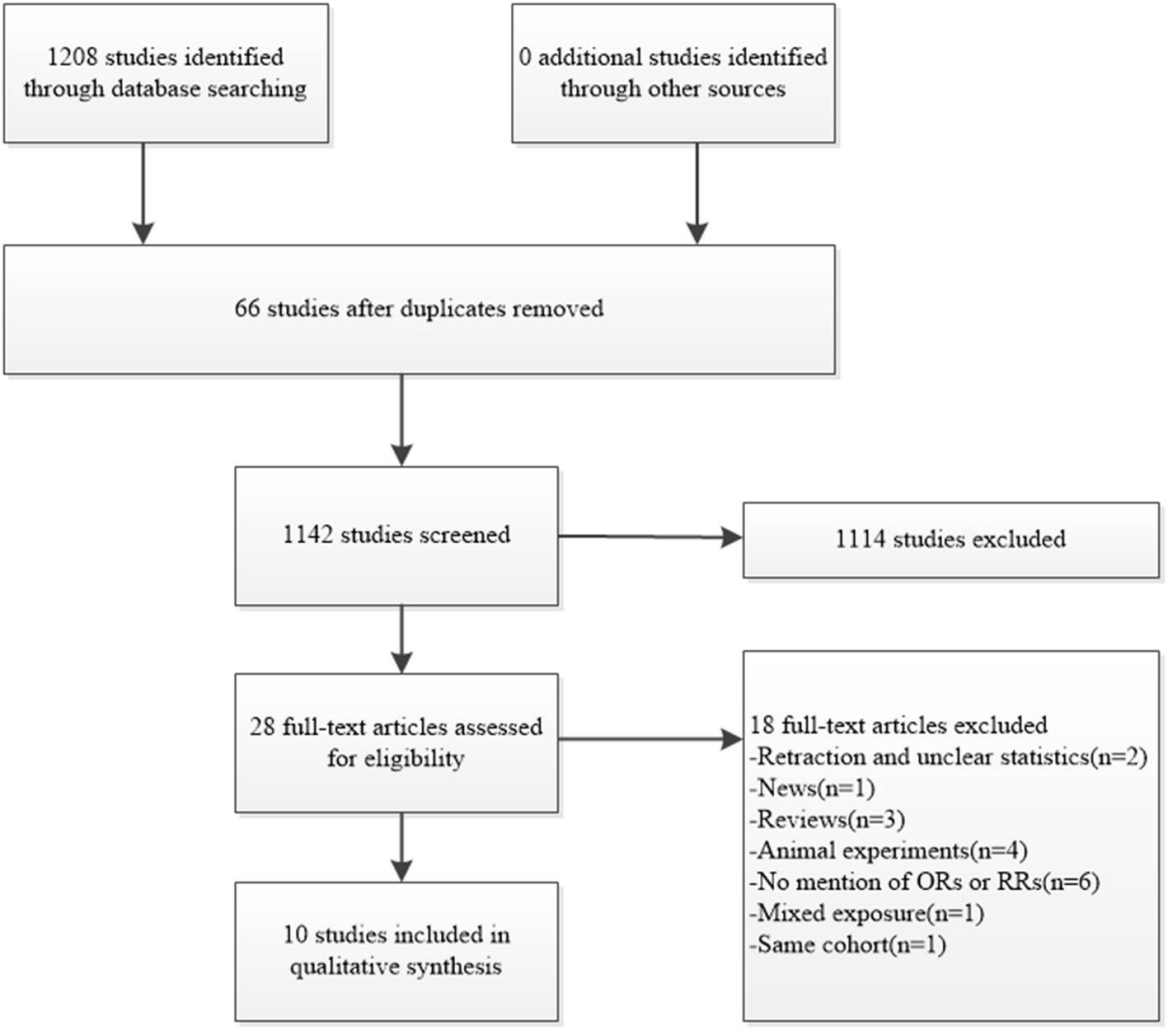
Flowchart for the search on cadmium exposure and risk of GDM.

After searching the mentioned databases, 1,208 articles were identified as eligible in total, and 66 were excluded after finding duplicates. A total of 1,142 articles were then further screened, and 1,114 were excluded for different reasons, such as unrelated context, meeting abstract, or animal studies. The full texts of the remaining 28 articles were screened further. Among them, 1 news article, 1 retraction, 3 reviews, and 4 animal experiments were excluded. A total of 6 studies did not mention the ORs or RRs, and 1 study focused on mixed exposure. One study was then excluded for unclear statistics (12). We also attempted to send an e-mail to the author for data clarification but received no reply. Another two studies were from the same cohort, and we therefore chose the most recent one (13). Finally, we included the remaining 10 studies in the meta-analysis on the connection between cadmium exposure and the risk of GDM (Figure 1). Some studies focusing on this issue have found significant evidence to prove the relationship between cadmium and the risk of GDM (14–17), while others have found negative results (13, 18–22).

Among the 10 studies, six were cohort studies, while four were case-control studies. Seven studies were conducted in China, one in the USA, one in Japan, and one in Canada. Five of them measured the concentrations of cadmium in urine samples, three in blood samples, one in meconium samples, and one in hair samples. The quality scores of all the included studies ranged from 7 to 9 and were regarded as high quality. The eligible studies were published from 2015 to 2021, and 32,000 pregnant women were enrolled in total. The characteristics and NOS scores of the 10 studies are described in Table 1.

**Table 1:** Characteristics of eligible studies investigating the association between cadmium exposure and gestational diabetes mellitus.

**References**	**Time period**	**Location**	**Design**	**Diagnostic criteria for GDM**	**Sample type**	**Time of sample collection**	**Sample size**	**Concentration of cadmium**	**OR/RR (95%CI)**	**Covariates adjusted**	**NOS**
Romano et al. (18)	2009–2017	USA	Cohort	ACOG	Urine	24–28 w	623	Normal	0.86 (0.51–1.44)	A, B, C, D, G, H	7
								0.06 (0.03–0.13) μg/L^a^			
								IGT/GDM:			
								0.09 (0.05–0.16) μg/L^a^			
Wang et al. (19)	2009–2017	China	Case-control	IADPSG	Blood	At delivery	1,552	Low (<0.69) μg/L	Low: 1	B, D, F, I, J, K	8
								Middle (0.69–3.43) μg/L	Middle: 1.01 (0.78–1.31)		
								High (>3.43) μg/L	High: 0.83 (0.64–1.08)		
Oguri et al. (20)	2011–2014	Japan	Cohort	JSOG and JAOG	Blood	22–28 w	16,076	①≤ 0.50 ng/g	Nulliparous:①1	A, B, C, L, M, Q, R, S	9
								②0.51–1.00 ng/g	②0.86 (0.53-1.38)		
								③1.01–1.50 ng/g	③0.87 (0.45-1.66)		
								④≥1.51 ng/g	④0.81 (0.30-2.20)		
									Parous:①1		
									②1.26 (0.88-1.81)		
									③1.37 (0.87-2.16)		
									④0.60 (0.27-1.34)		
Liu et al. (21)	2013–2016	China	Cohort	IADPSG	Urine	13 w	2,026	Low (<0.51) μg/L	Low: 1	A, B, C, F, M, N, P, T	8
								Medium (0.51–0.86) μg/L	Medium: 1.04 (0.74-1.44)		
								High (≥0.86) μg/L	High: 1.36 (0.98–1.90)		
Xing et al. (14)	2012–2014	China	Cohort	IADPSG	Urine	3 days before delivery	6,837	Q1 (<0.40) μg/g	Q1: 1	A, B, F, M, N	8
								Q2 (0.40–0.58) μg/g	Q2: 1.21 (0.97–1.50)		
								Q3 (0.58–0.85) μg/g	Q3: 1.24 (1.00–1.53)		
								Q4 (≥0.85) μg/g	Q4: 1.30 (1.05–1.61)		
Peng et al. (16)	2012	China	Case-control	IADPSG	Meconium	First 2 postnatal days	327	Normal	Q1: 1	A, B, E, F, P, U	7
								4.10 (1.47–11.32) ng/L	Q2: 3.07 (0.69–13.74)		
								GDM	Q3: 16.87 (4.19–67.86)		
								9.41 (5.59–15.23) ng/L	Q4: 11.95 (2.97–48.04)		
Shapiro et al. (15)	2008–2011	Canada	Cohort	CDA	Blood	First-trimester	1,181	Q1 (0.0–0.1) μg/L	Q1: 1	A, B, C, N, O, V	7
								Q2 (0.2–0.2) μg/L	Q2: 2.1 (0.8-5.4)		
								Q3 (0.2–0.3) μg/L	Q3: 1.4 (0.5-3.9)		
								Q4 (0.3–5.1) μg/L	Q4: 2.5 (1.0-6.4)		
Wang et al. (13)	2014–2016	China	Cohort	IADPSG	Urine	13 w	2,090	T1 (≤0.65) μg/g	T1: 1	A, B, C, D, F, J, P, T, W	8
								T2 (0.65–1.10) μg/g	T2: 0.94 (0.70, 1.26)		
								T3 (?1.10) μg/g	T3: 0.86 (0.62, 1.19)		
Li et al. (17)	2013–2016	China	Case-control	IADPSG	Urine	13 w	610	T1 (<0.41) μg/L	T1: 1.00	A, B, C, F, N, X	8
								T2 (0.41–0.83) μg/L	T2: 2.37 (1.33, 4.24)		
								T3 (≥0.83) μg/L	T3: 2.28 (1.25, 4.16)		
Jia et al. (22)	2017	China	Case-control	IADPSG	Hair	First-trimester	678	Normal	Low: 1.00	A, B, C, F, I, M, W, Y	8
								0.015 (0.010–0.023) μg/g	Medium: 1.10 (0.91–1.33)		
								GDM	High: 1.03 (0.85–1.24)		
								0.015 (0.010–0.025) μg/g			

a *Data are presented as mean (interquartile range, IQR). ACOG, American College of Obstetricians and Gynecologists; JSOG and JAOG, Japan Society of Obstetrics and Gynecology and Japan Association of Obstetricians and Gynecologists; IADPSG, International Association of Diabetes and Pregnancy Study Group; CDA, Canada Diabetes Association; Q1, the 1st quartile; Q2, the 2nd quartile; Q3, the 3rd quartile; Q4, the 4th quartile; T1, the 1st tertile; T2, the 2nd tertile; T3, the 3rd tertile; IOR, Inter-quartile range, GDM, gestational diabetes mellitus; IGT, Impaired Glucose Tolerance; A, age; B, BMI; C, smoking; D, pregnancy weight gain; E, gravidity; F, parity; G, creatinine (mg/dL); H, gestational age at glucose testing; I, physical activities; J, family history of diabetes; K, month of conception; L, other maternal history of GDM (parous group only); M, pregnancy-induced hypertension; N, education; O, race; P, fetal sex; Q, carbohydrate intake (g/kcal/day); R, alcohol consumption; S, dyslipidemia; T, total arsenic level; U, HBV infection (HBsAg positive); V, household income; W, employment status; X, urinary total arsenic, chromium, manganese, and zinc; Y, hair dye*.

### Combined Effect Evaluation

**Figure 2:**
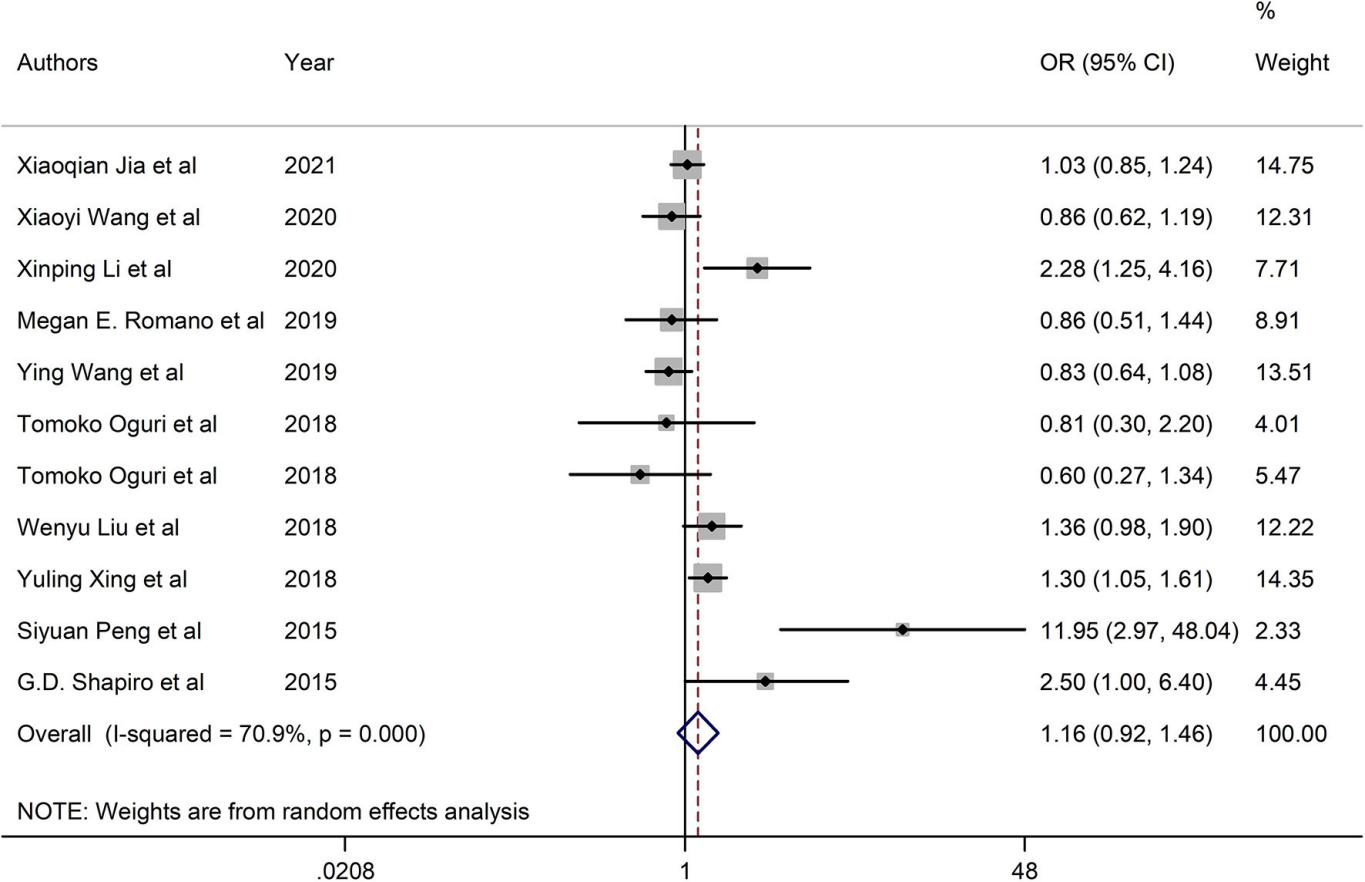
Forest plots of associations between cadmium exposure and the risk of GDM.

The pooled OR of cadmium exposure was 1.16 (95% CI = 0.92, 1.46) in the random-effects model due to the high heterogeneity, revealing that cadmium exposure had no significant association with the increased risk of GDM (Figure 2).

### Sensitivity Analyses and Publication Bias

**Figure 3:**
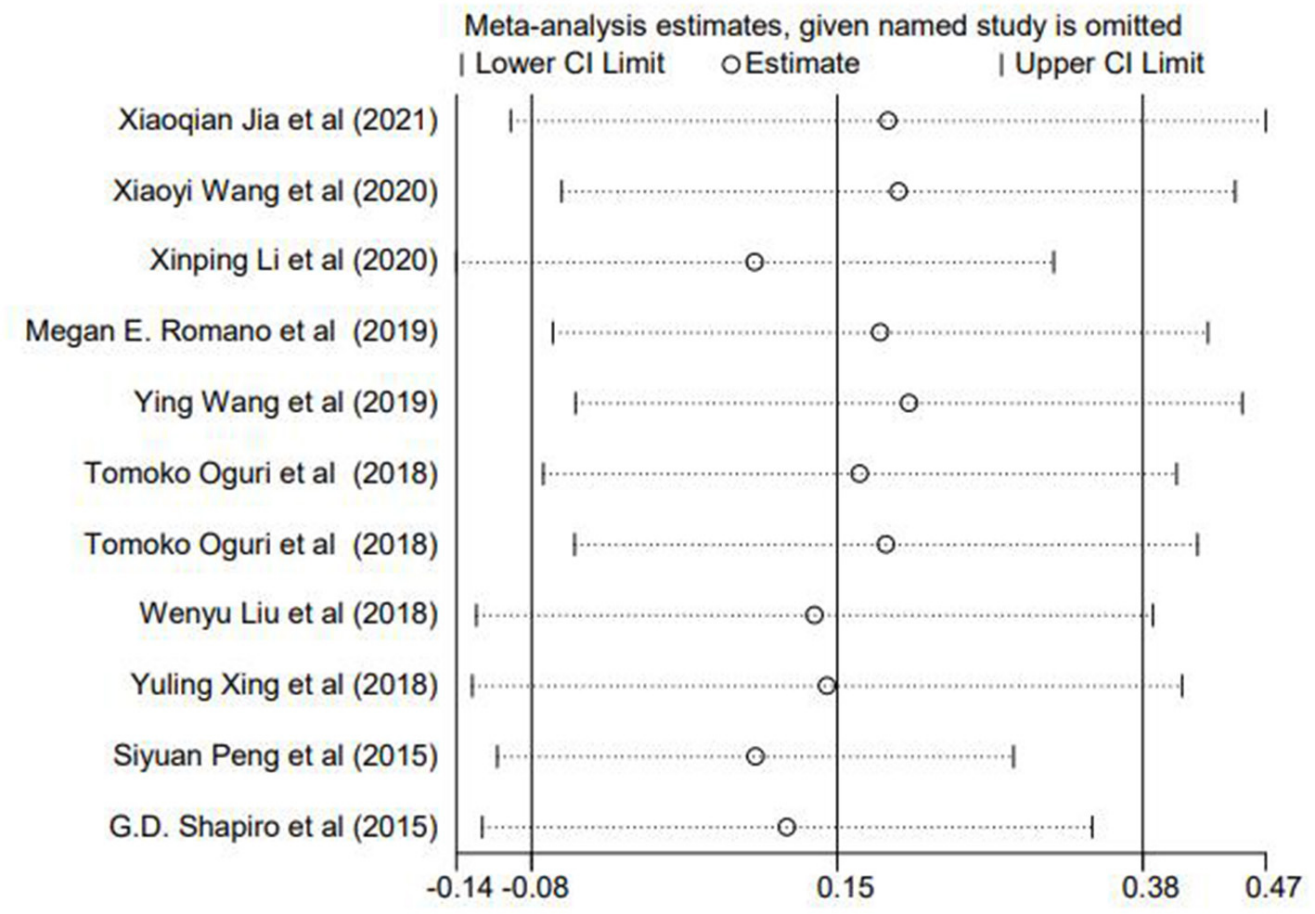
Sensitivity analyses after omitting each study.

To evaluate whether the findings was changed by excluding each study, a sensitivity analysis was performed. As shown in Figure 3, no single study had a significant effect on the summary ORs, thereby supporting the stableness of our results.

**Figure 4:**
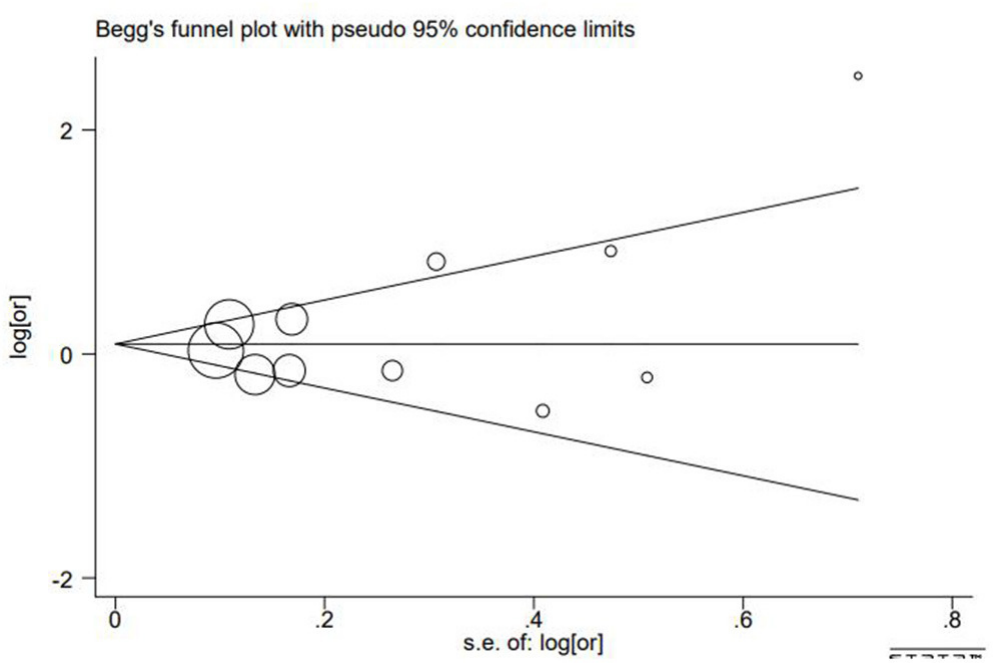
Funnel plots of associations between cadmium exposure and the risk of GDM.

Egger's test and Begg's test were used to calculate the publication bias of these studies. We found no publication bias according to the results (*P*_Egger_ = 0.316, *P*_Begg_ = 0.213, Figure 4).

### Meta-Regression Analysis and Subgroup Analysis

We performed meta-regression analysis to assess the sources of heterogeneity, including publication year, geographic region, design, sample type, time of sample collection, sample size, and NOS score. As shown in Table 2, publication year contributed to the high heterogeneity (*P* = 0.034). However, the source of heterogeneity was not observed among other factors (all *P* > 0.05).

**Table 2:** The result of meta-regression analysis.

**Covariates**	**Coefficient**	**95%CI**	***P***
Publication year	−0.3421	(−0.636, −0.049)	0.034
Geographic region	−0.9730	(−2.050, 0.104)	0.064
Design	−1.3313	(−2.773, 0.111)	0.061
Sample type	−0.1621	(−0.710, 0.386)	0.416
Time of sample collection	−0.8045	(−1.698, 0.089)	0.064
Sample size	0.0003	(−0.000,0.001)	0.096
NOS	−2.1129	(−4.797,0.571)	0.087

*CI, confidence interval*.

In subgroup analysis, when stratified by publication year, the summary OR of nine studies with publication year before 2016 was 4.05 (95% CI = 1.87–8.76, *P* < 0.001). When stratified by study design, we found a borderline increased risk of gestational diabetes due to cadmium exposure in cohort studies (OR = 1.15, 95% CI = 0.99–1.33, *P* = 0.061). Subgroup analyses by geographic region, diagnosis criteria, sample type, sample size, NOS score, and time of collection were not statistically significant (all *P* > 0.05, Table 3).

**Table 3:** Results of the subgroup analysis for the associations of cadmium exposure with GDM risk.

**Variables**	***N***	***P^a^***	**OR (95% CI)**
All studies	11	<0.001	1.16 (0.92, 1.46)
**Publication year**			
Before 2016	9	0.067	4.05 (1.87–8.76)
After 2016	2	0.011	1.06 (0.88–1.28)
**Geographic region**			
Asia	9	<0.001	1.15 (0.90, 1.47)
North America	2	0.049	1.36(0.48, 3.84)
**Design**			
Cohort	7	0.058	1.15 (0.99, 1.33)
Case-control	4	<0.001	1.48 (0.88, 2.49)
**Diagnosis criteria**			
ACOG	1	–	0.86 (0.51, 1.45)
IADPSG	7	<0.001	1.23 (0.94, 1.60)
JSOG and HAOG	2	0.645	0.68 (0.36, 1.26)
CDA	1	–	2.50 (0.99, 6.32)
**Sample type**			
Urine	5	0.026	1.21 (0.92, 1.58)
Blood	4	0.115	0.86 (0.68, 1.09)
Meconium	1	–	11.95 (2.97, 48.06)
Hair	1	–	1,03 (0.85, 1.24)
**Sample size**			
≤1,000	4	<0.001	1.67(0.87, 3.22)
>1,000	7	0.013	1.06 (0.82, 1.36)
**NOS score**			
≤ 7	3	0.001	2.60 (0.66, 10.31)
>7	8	0.008	1.08 (0.88, 1.32)
**Time of collection**			
The first trimester	5	0.012	1.27 (0.94, 1.73)
The second trimester	3	0.758	0.78 (0.52, 1.16)
The perinatal period	3	<0.001	1.47 (0.78, 2.77)

a *Tests for heterogeneity*.

## Discussion

Overall, our manuscript shows no statistically significant association between cadmium exposure and the risk of GDM. However, the negative results should be interpreted with caution for several reasons.

In subgroup analyses, we observed a borderline increased risk of disease in cohort studies. Cohort studies are conducted to determine the causal association between initial participant exposure characteristics and diseases, while case-control studies can only prove the potential relationship between exposure characteristics and diseases. Cohort studies have several strengths when compared with case-control studies, such as a large number of participants and relatively complete data. Moreover, studies enrolling <1,000 participants are more than 50% in our meta-analysis. Meta-analyses may be inaccurate because of the smaller size of the included studies and the low number of participants, which may result in more random errors (23). Several studies have studied the role of cadmium exposure in the development of GDM at present, which has led to an insufficient number of included studies in our meta-analysis. At last, the result of meta-regression showed that the time of publication contributed to the high heterogeneity. The methods of detecting cadmium are becoming more and more accurate and multiple, and the design may be more reasonable according to the previous studies. Notably, the measurements and samples for cadmium exposure were variable among these included studies. Debate still remains as to which type of sample reflects the cadmium burden. Different samples can be used to reflect the chemical exposure of a particular pregnancy time period. Additionally, it is crucial to consider all available analytes to determine the actual cadmium burden of the body during pregnancy (24). All studies selected in our meta-analysis adopted only one type of sample to measure the cadmium burden of pregnant women, which may not correctly reflect the total body burden of cadmium. As a result, the association between cadmium exposure and the risk of GDM may have been influenced by different or even improper measurements. We also conducted subgroup analyses according to the different processes of pregnancy due to the pathophysiology of GDM. It has been proven that there is decreased insulin sensitivity before conception in pregnant women with GDM, which further decreases during pregnancy (25, 26). This means that potential changes in the pathophysiology of GDM may appear earlier than the first trimester of pregnancy. Although the development of GDM results from changes in pathophysiology during the full process of pregnancy, changes in early life are still more important. However, all the included studies in our meta-analysis evaluated cadmium exposure after conception. Five studies measured the cadmium burden in the first trimester, three in the second trimester, and three in the perinatal period, and no statistically significant association was found.

In addition, some emerging factors are associated with GDM, such as fetal sex and toxic metals including arsenic and antimony (27, 28). As mentioned in the study done by Liu, women carrying male fetuses tend to develop insulin resistance when compared with those carrying female fetuses. Additionally, unknown or unmeasured co-exposures also exist in our environment, which could result in an increased risk of GDM. Although, the studies took the possible confounding factors related to GDM into consideration, these limitations possibly affected the results of our meta-analysis. Nevertheless, not all included studies evaluated the risk with models adjusted by exposure to other metals. Moreover, the adjusted confounding factors were variable among these studies. Multiple factors were verified to be connected with GDM, such as BMI, smoking, family history of diabetes, and alcohol consumption.

Although, we found no statistically significant association between cadmium exposure and GDM, it is still possible that cadmium plays an important role in the development of GDM. Cadmium exposure has been linked to inflammation and oxidative stress, and it up-regulates the expression of tumor necrosis factor (TNF) and different chemokines in various cell types (10). TNF activates a signaling pathway that increases the levels of sphingomyelinase and ceramides, which disrupt the insulin signaling pathway (29). The potential effect of cadmium on type 2 diabetes has been shown in several studies. Some experiments found that cadmium exposure can destroy the function of β-cells and cause insulin resistance (9). However, the role of cadmium exposure in GDM remains unclear. Moreover, we performed this analysis by using the highest vs. the lowest exposure group rather than the continuous exposure group. Therefore, the concentrations of cadmium were variable among these studies, and participants in studies with high concentrations of cadmium seemed to have a higher risk of GDM. As a result, other studies with relatively lower levels of cadmium could have obtained negative effects. Prospectively, more epidemiological proof and experimental efforts are urgently needed.

Our meta-analysis has several strengths. First, our meta-analysis is the first to explore the relationship between the risk of GDM and cadmium exposure. Second, the included studies were limited to English papers and Chinese papers, the latter of which could be more objective and representative for elaborating the potential effect of cadmium on GDM. Third, sub-analyses were conducted to further explore the associations and determine the source of heterogeneity, and the null results may have been due to the limited number of participants. Nevertheless, our study has several limitations. The small sample size may have resulted in random errors in our meta-analysis due to the relatively less attention given to GDM and cadmium exposure. Three studies had NOS scores ≤ 7, indicating potential defects in methods or study design, which may have affected the accuracy of our meta-analysis. Third, the accuracy of the analysis was affected by the high heterogeneity and inconsistent adjusted confounding factors.

## Conclusion

In conclusion, we found no statistically significant association between cadmium exposure and the risk of GDM. More studies with larger sample sizes and detailed experiments should be conducted to further explore the associations and identify the role of cadmium exposure in the mechanism of GDM.

## Data Availability Statement

The original contributions presented in the study are included in the article/Supplementary Material, further inquiries can be directed to the corresponding author/s.

## Author Contributions

YL and KX designed the study. YL and TL searched for and evaluate the included studies. ZS and KX contributed to the funding acquisition and manuscript editing. YL and JX wrote the manuscript. KX analyzed data. All authors contributed to the article and approved the submitted version.

## Funding

This research was supported by the National Natural Science Foundation of China (81971410 and 81571458), Postdoctoral Research Funding Program of Jiangsu Province (2020Z146), and State Key Laboratory of Reproductive Medicine (SKLRM-UC201901 and SKLRM-UC201902).

## Conflict of Interest

The authors declare that the research was conducted in the absence of any commercial or financial relationships that could be construed as a potential conflict of interest.

## Publisher's Note

All claims expressed in this article are solely those of the authors and do not necessarily represent those of their affiliated organizations, or those of the publisher, the editors and the reviewers. Any product that may be evaluated in this article, or claim that may be made by its manufacturer, is not guaranteed or endorsed by the publisher.
