# Supplementary material for: The Association Between Cadmium Exposure and Gestational Diabetes Mellitus: A Systematic Review and Meta-Analysis
Source: Front Public Health. 2022 Feb 10;9:555539. doi: 10.3389/fpubh.2021.555539 (PMC8866184; doi:10.3389/fpubh.2021.555539)

Supplementary materials

**Supplementary Table 1. The participants, interventions, comparisons, outcome, and study design (PICOS) strategy in our study.**

| Population Pregnant women with GDM |
| --- |
| Intervention Cadmium exposure |
| Control Healthy pregnant women or pregnant women without GDM |
| Outcomes The association with cadmium exposure and GDM |
| Study design Case-control, cohort |

**Supplementary Figure 1. The search strategy of five databases in our meta-analysis.**


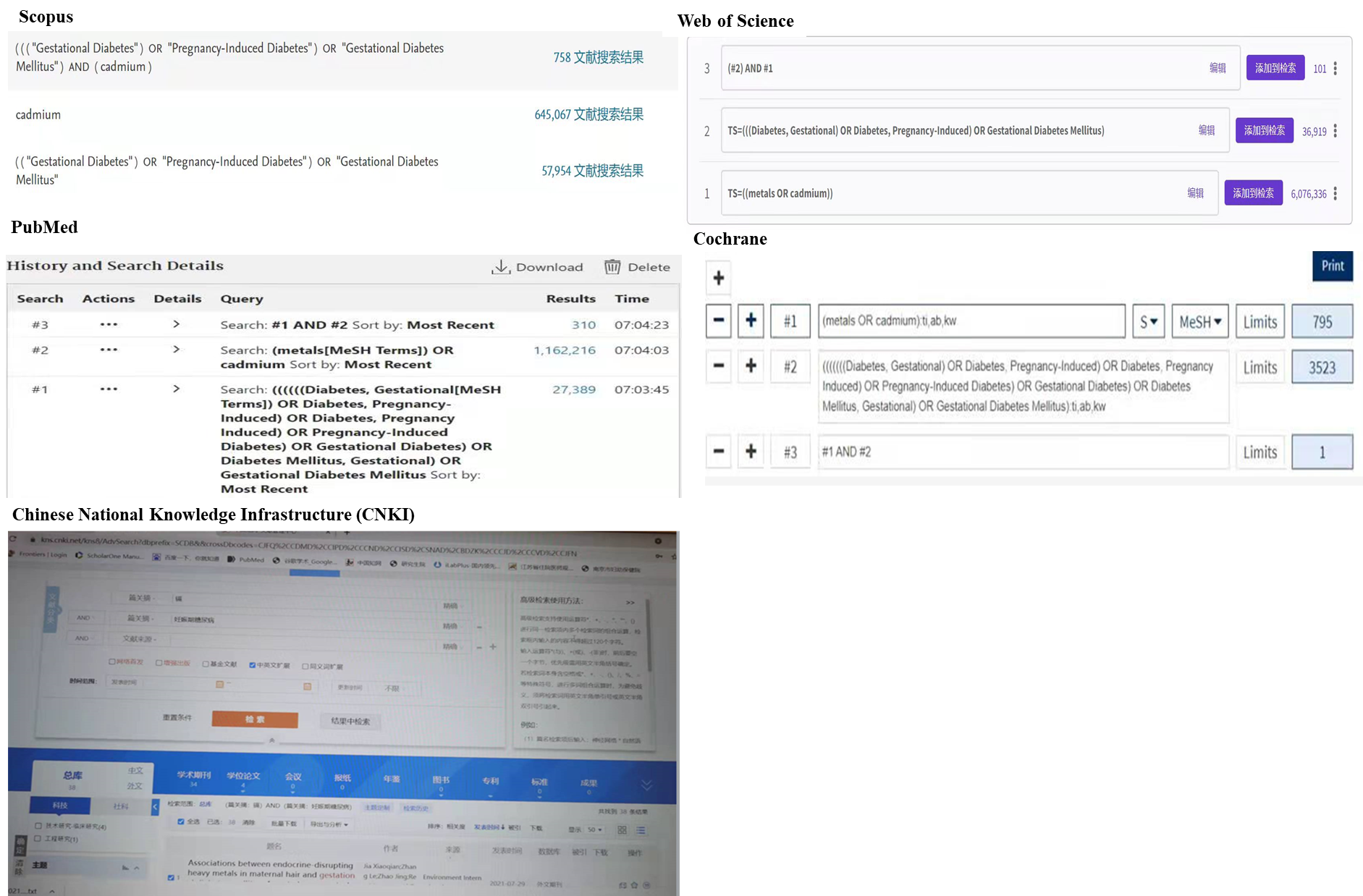

Supplement: Supplementary file 1 [file Data_Sheet_1.doc]
